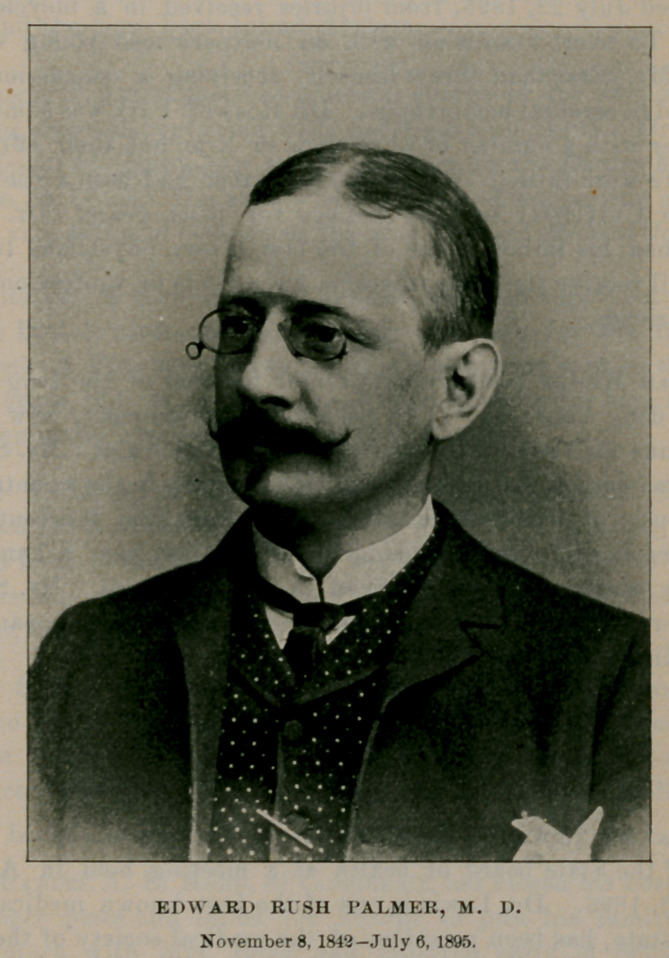# Dr. Edward Rush Palmer

**Published:** 1895-08

**Authors:** 


### Obituary.

---

Dr. Edward Rush Palmer, of Louisville, Ky., died on the night of July 5-6, 1895, from the effects of an injury received in a bicycle collision while riding on the Third street boulevard in the city of his home. The accident occurred late in the evening of July 5th, by which he was hurled headlong against the curbstone. He almost immediately became unconscious from a fracture of the base of the skull, and was taken to the Norton Infirmary, where he died at 12.30 A. M., July 6th.

Edward Rush Palmer was born in Woodstock, Vt., November 18, 1842. His father, Dr. Benjamin Rush Palmer, removed with his family to Kentucky when his only son, Edward, was eight years old, where he became professor of surgery in the University of Louisville. Edward R. graduated in medicine in 1864 from this same school and then made haste to enter the medical corps of the army. He served in military hospitals in Louisville and Lebanon, and at the end of the war returned to his home, where he entered upon the general practice of medicine. About ten years ago he abandoned his large family practice to devote himself to the specialty of genito-urinary surgery, in which he became celebrated. In 1868, he was chosen professor of physiology in his Alma Mater and held a chair in that institution until he died. In 1893, he was elected president of the American Association of Genito-urinary Surgeons, and attended its last meeting at Niagara Falls, May 29–30, 1895. He was also a member of the American Medical Association, of the Mississippi Valley Medical Association, of the Kentucky State Medical Society, of the Medico-chirurgical Society of Louisville, and was president and one of the founders of the Surgical Society of Louisville.

It is not often that we are called upon to record such a painful incident in these columns. Dr. Palmer was a man who took great enjoyment in life and though turned well into the fifties he was as fresh and vivacious as a boy in his 'teens while yet strong in his well-ripened manhood. He had everything to live for and every expectation of longevity. He was surrounded by an interesting family, consisting of a lovely wife, a charming daughter, well grown into young womanhood, and two manly sons, both graduates of Princeton and who expect to take their medical degrees next year. His accomplishments were many and versatile. A lover of art, a student of nature, cultivated in music and a clever conversationalist, he was, at once, an attractive man, a delightful companion, an earnest friend, whose loss will be felt for many years in society as well as in every other walk which men of such rare attainments grace by their presence.

EDWARD RUSH PALMER, M. D.

November 8, 1842—July 6, 1895.

And Dr. Palmer was a physician singularly gifted in professional acquirements. A teacher of conspicuous excellence, a general practitioner of medicine for many years of unusual skill and judgment, and more recently a specialist of national fame enjoying the confidence of a large clientele and a widely distributed group of his professional brethren, it is difficult to appreciate how a man and a physician could have been more charmingly surrounded with everything that goes to make life delightful than Edward

Rush Palmer, whose spirit went out with a flash on that fateful July evening and who died lamented by every person who knew him.

## Figures and Tables

**Figure f1:**